# Colloidal Manganese Salt Improves the Efficacy of *Toxoplasma gondii* Inactivated Vaccine in Mice

**DOI:** 10.3390/vaccines13121230

**Published:** 2025-12-07

**Authors:** Chebing Huang, Shaoyuan Bai, Haiqiong Yu, Ming Pan, Zhaofeng Hou, Lizhi Fu, Siyang Huang

**Affiliations:** 1 Institute of Comparative Medicine, College of Veterinary Medicine, Yangzhou University, and Jiangsu Co-innovation Center for Prevention and Control of Important Animal Infectious Diseases and Zoonosis, and Jiangsu Key Laboratory of Zoonosis, Yangzhou 225009, China; mx120231058@stu.yzu.edu.cn (C.H.); shaoyuan2023@163.com (S.B.); panming@yzu.edu.cn (M.P.); zfhou@yzu.edu.cn (Z.H.); 2 Guangzhou Customs District Technology Center, Guangzhou 510623, China; 006829@yzu.edu.cn; 3 Chongqing Academy of Animal Sciences, Chongqing 402460, China; fulz@cqaa.cn

**Keywords:** *Toxoplasma gondii*, immune response, colloidal manganese salt, adjuvant, vaccine

## Abstract

Background/Objectives: Toxoplasmosis caused by *Toxoplasma gondii* still poses a serious threat to public health in most countries and regions. Currently, the lack of effective vaccines necessitates the urgent development of a safe and effective vaccine. Methods: In this study, we combined the inactivated *T. gondii* vaccine with a colloidal manganese salt (Mn jelly [MnJ]) adjuvant. Results: This triggered a powerful innate immunity, significantly increased the number of CD4^+^ and CD8^+^ T cells secreting interferon γ (IFN-γ) in mice, and enhanced the generation of CD8^+^ central memory T cells and CD8^+^ effector memory T cells. Compared to the control groups, mice in experimental groups produced more specific IgG, and produced high levels of IL-2, IL-12 and IFN-γ. The survival rate of mice in experimental groups reached 50%, while all control group mice died within 9 days during *T. gondii* acute infection. Furthermore, the burden of brain cysts in experimental group mice was also significantly reduced by 90.77% compared to the control group during chronic infection. Conclusions: These results suggested that the incorporation of an MnJ adjuvant significantly enhances the immunoprotective efficacy of inactivated *T. gondii* vaccine, positioning this formulation as a promising candidate for development against toxoplasmosis.

## 1. Introduction

*Toxoplasma gondii* (*T. gondii*) is an obligate intracellular parasitic protozoan that can infect almost all warm-blooded vertebrates. Most immunocompetent individuals are asymptomatic or mildly symptomatic, whereas immunocompromised patients are vulnerable to severe, potentially fatal disease [1]. Pregnant women infected with *T. gondii* can suffer from serious consequences such as premature birth and miscarriage, and it can also cause symptoms like deformities in newborns [2,3]. Currently, there is no widely recognized, safe and effective *T. gondii* vaccine approved for marketing by authoritative agencies worldwide [4].

Previous studies indicated that the inactivated *T. gondii* vaccine eliminates pathogen infectivity or pathogen viability through targeted inactivation, effectively eliminating infectivity while preserving critical immunogenic epitopes to induce humoral immune responses [5,6]. This vaccine platform demonstrates distinct advantages over alternatives, including enhanced biosafety profiles, simplified cold-chain logistics, scalable production capabilities, and cost-effective manufacturing processes [7]. However, its immunogenic limitations, particularly inadequate cellular immune activation required for intracellular parasite clearance, significantly constrain protective efficacy when administered alone [8]. Therefore, we aim to utilize adjuvants to enhance the activation of cellular immunity by inactivated vaccines.

Traditional aluminum adjuvants are widely used; they can enhance humoral immunity but have a weak ability to activate cellular immunity, failing to meet the protective needs against intracellular pathogens [9]. Oil-emulsion adjuvants can sustain antigen release, activate immune cells, and indirectly promote Th1-type cellular immunity, yet they may induce inflammatory reactions at the injection site [10]. Manganese ions (Mn^2+^) act as second messengers, synergistically promoting the expression of type I interferons and pro-inflammatory cytokines by activating the cGAS-STING nucleic acid recognition pathway and RIG-I/MDA5 signaling axis [11], which enhances dendritic cell maturation, antigen-presenting efficiency, and Th1-type immune responses, thereby improving the cytotoxic activity of T lymphocytes [12]. Given that Mn^2+^ induces robust type I interferon (IFN) production and NLRP3 inflammasome activation, both of which are closely associated with enhanced adjuvant activity, studies have confirmed that the colloidal manganese salt (Mn jelly [MnJ]) as an adjuvant exhibits significant application potential in viral vaccines [13]. It notably improves the immunogenic efficiency of inactivated viral vaccines, reduces antigen dosage requirements, and enhances cross-protection against heterologous viral strains [14]. Preclinical studies have shown that the MnJ adjuvant has been successfully applied in the industrial production of inactivated avian influenza vaccines for pets and subunit vaccines against canine parvovirus, with thermal stability significantly superior to aluminum salt adjuvants [13]. However, there is no information about MnJ adjuvant combined with vaccines against parasites. This study innovatively proposes a strategy of combining manganese adjuvant with inactivated *T. gondii* vaccine, expecting that the addition of manganese adjuvant will enable the inactivated vaccine to enhance cellular immunity against *T. gondii*, providing novel strategies for toxoplasmosis control.

## 2. Materials and Methods

### 2.1. Mice, Parasites, Adjuvant

Six-week-old specific pathogen-free (SPF) ICR mice were obtained from the Laboratory Animal Research Center affiliated with Yangzhou University. All experimental protocols adhered strictly to the Guidelines for the Care and Use of Laboratory Animals and were granted ethical approval by the Institutional Animal Care and Use Committee (IACUC) of Yangzhou University (Approval No. 202302012; 4 March 2023). Vero cells were cultured in 25 cm^2^ culture flasks (Thermo Scientific™, Waltham, MA, USA) with Dulbecco’s modified Eagle medium (DMEM; Gibco™, Grand Island, NY, USA) supplemented with 5% fetal bovine serum (FBS), 100 U/mL penicillin, and 10 mg/mL streptomycin, incubated in a 37 °C incubator with 5%CO_2_. RH-strain *T. gondii* tachyzoites were propagated in Vero following established protocols. The MnJ used in this study was kindly provided by Professor Zhengfan Jiang. The inactivated *T. gondii* vaccine was preliminarily prepared by the laboratory. We utilized a novel, verified low-temperature inactivation method, distinguished from traditional inactivation approaches. Specifically, cultured RH tachyzoites were centrifuged at 700× *g* and resuspended in PBS. They were subsequently incubated at 37 °C for 24 h and maintained at 4 °C for 20 days. Prior to use, the concentration of the inactivated vaccine was adjusted to 10^6^ inactivated tachyzoites [15].

### 2.2. Immunization and Challenge Experiment

**Figure 1:**
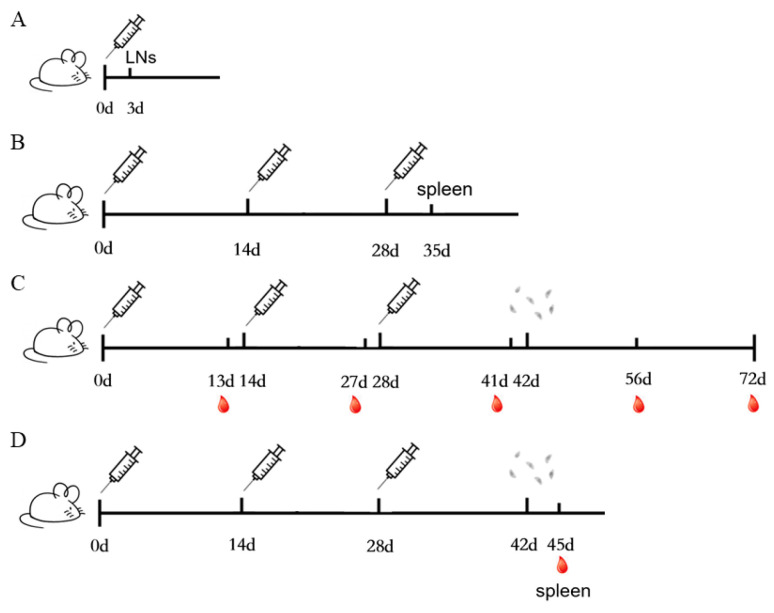
Immunization method. (**A**) The immunization schedule used in Section 3.1. (**B**) The immunization schedule used in Section 3.2, Section 3.3 and Section 3.4. (**C**) The immunization schedule used in Section 3.5 and Section 3.6. (**D**) The immunization schedule used in Section 3.7.

One hundred twenty-two mice were randomly divided into four groups: A: PBS; B: *T. gondii* inactivated vaccines (VAC); C: MnJ adjuvant; D: VAC + MnJ adjuvant. Each mouse in group D was immunized intramuscularly (*i.m.*) with 100 μL of inactivated vaccine and MnJ adjuvant (1 mg/mL), the inactivated vaccines containing 10^6^ inactivated tachyzoites, and mice in the other groups were immunized with PBS, VAC or MnJ adjuvant as controls, respectively; the immunization method is shown in Figure 1A–D. Blood samples were collected one day before each immunization for analysis. Each mouse ultimately yielded approximately 100 μL of serum. Lymph node samples (inguinal lymph nodes, popliteal lymph nodes, and iliac lymph nodes) were collected three days after the first immunization for analysis. Spleen samples were collected seven days after the last immunization for analysis. Challenge process: Two weeks after the last immunization, 10 mice from each group were intraperitoneally injected with 1 × 10^2^ tachyzoites of the RH and the survival times were recorded daily until 30 days post-infection (dpi). In parallel, another 10 mice were given 20 brain cysts orally and euthanized at 30 dpi; the brains of the mice were collected and ground into homogenized suspensions, and the number of cysts was calculated by microscopic observation.

### 2.3. Flow Cytometry

The mice were sacrificed by cervical dislocation; the subcutaneous tissue was exposed by blunt dissection. By identifying the intersection points of the three major subcutaneous blood vessels in the inguinal region, popliteal region, and iliac vessel region, the inguinal lymph nodes, popliteal lymph nodes, and iliac lymph nodes were accurately located and isolated. Meanwhile, the spleen tissue was completely removed. All the excised tissue samples were immediately stored in pre-cooled phosphate-buffered saline (PBS) containing 0.5% fetal bovine serum (FBS). A single-cell suspension was prepared, counted, stained, and then loaded onto the machine for analysis. DCs: Cellular debris was excluded using a forward scatter area (FSC-A) vs. side scatter area (SSC-A) dot plot to obtain total cells; single cells were gated on a forward scatter height (FSC-H) vs. forward scatter width (FSC-W) dot plot; and CD11c^+^MHC-II^+^ and CD11c^+^CD80^+^ cell populations were gated, with thresholds set by isotype controls. IFN-γ-secreting T cells: Debris exclusion via FSC-A vs. SSC-A; T cells were gated as CD3^+^ cells on an FSC-A vs. CD3 dot plot; CD4^+^ T cells and CD8^+^ T cells were further discriminated within the CD3^+^ population on a CD4 vs. CD8 dot plot. IFN-γ^+^ cells were identified within CD3^+^ CD4^+^ T cells, with thresholds set by isotype controls. Memory T cells: Debris exclusion via FSC-A vs. SSC-A; T cells were gated as CD3^+^ cells on FSC-A vs. CD3 dot plot; CD4^+^ T cells and CD8^+^ T cells were further discriminated within the CD3^+^ population on a CD4 vs. CD8 dot plot. CD4 naive: CD4^+^ CD44^lo^CD62L^hi^, CD4 C.M.: CD4^+^ CD44^hi^CD62L^hi^, CD4 E.M.: CD4^+^ CD44^hi^CD62L^lo^ cells were identified within CD3^+^ CD4^+^ T cells, with thresholds set by isotype controls.

### 2.4. Quantitative Real-Time PCR (qRT-PCR)

Total RNA was isolated using TRIzol reagent (Invitrogen, Karlsruhe, Germany), treated with DNase to eliminate potential DNA contamination, and then transcribed into complementary DNA (cDNA) by means of FSQ-201 ReverTra Ace (Toyobo, Osaka, Japan). For quantitative real-time polymerase chain reaction (qRT-PCR) analysis, SYBR green (Bio-Rad, Hercules, CA, USA) was employed as the fluorescent dye, and the reaction was performed on an Applied Biosystems 7300 (Applied Biosystems, Foster City, CA, USA). Primers used in this study are listed in Table 1.

### 2.5. Enzyme-Linked Immunosorbent Assay (ELISA)

Blood samples were gathered and allowed to stand at room temperature for 2 h, after which they were transferred to 4 °C for overnight storage. Sera were isolated via centrifugation at 700× *g* for 10 min and preserved at −20 °C. Serum derived from mice administered with PBS served as negative controls.

Total immunoglobulin G (IgG) content and subclasses of IgG antibodies IgG1 and IgG2a were tested by ELISA. Toxoplasma lysate antigen (TLA) antigen was coated overnight at 4 °C; diluted mouse serum was added and incubated at 37 °C; horseradish peroxidase (HRP)-conjugated anti-mouse IgG was used for detection; substrate was added for reaction; and OD values were measured using a microplate reader [16]. Splenocytes were seeded in 96-well plates and stimulated with 10 μg/mL TLA and culture supernatants were collected to detect IL-2, IL-10, IL-12 and IFN-γ by ELISA according to the manufacturer’s instructions (Mlbio, Shanghai, China).

### 2.6. Cell Counting Kit-8 Assay

The collected splenocytes were treated with red blood cell lysate. Subsequently, the splenocytes were seeded into 96-well plates at a density of 1 × 10^6^ cells per well and cultured in DMEM medium supplemented with 100 μg/mL streptomycin/penicillin and 10% fetal bovine serum. The splenocytes were stimulated with 10 μg/mL TLA, 7.5 μg/mL Concanavalin A (ConA) (positive control), and the culture medium (negative control) and then incubated in an environment of 37 °C and 5% CO_2_ for 72 h. Subsequently, 50 μL of CCK-8 solution was introduced into each well, followed by an additional 4 h incubation period. The proliferative activity was assessed via determination of the optical density (OD) values at 450 nm using an ELISA reader (Bio-Tek EL × 800, Winooski, VT, USA). The splenocyte stimulation index (SI) was computed as the quotient of the mean absorbance of TLA-treated samples to that of the negative control groups.

### 2.7. Statistical Analysis

Data were expressed as mean ± standard deviation (SD). All statistical analyses were conducted using GraphPad Prism (version 8.3.0; GraphPad Software). Differences among the groups, such as those of antibody responses and cytokine levels, were assessed using a one-way analysis of variance (ANOVA). A *p*-value below 0.05 was deemed statistically significant. Significance levels were denoted as follows: * *p* < 0.05, ** *p* < 0.01, *** *p* < 0.001. ‘ns’ indicates a lack of significance.

## 3. Results

### 3.1. MnJ Adjuvant Promoted DC Maturation

**Figure 2:**
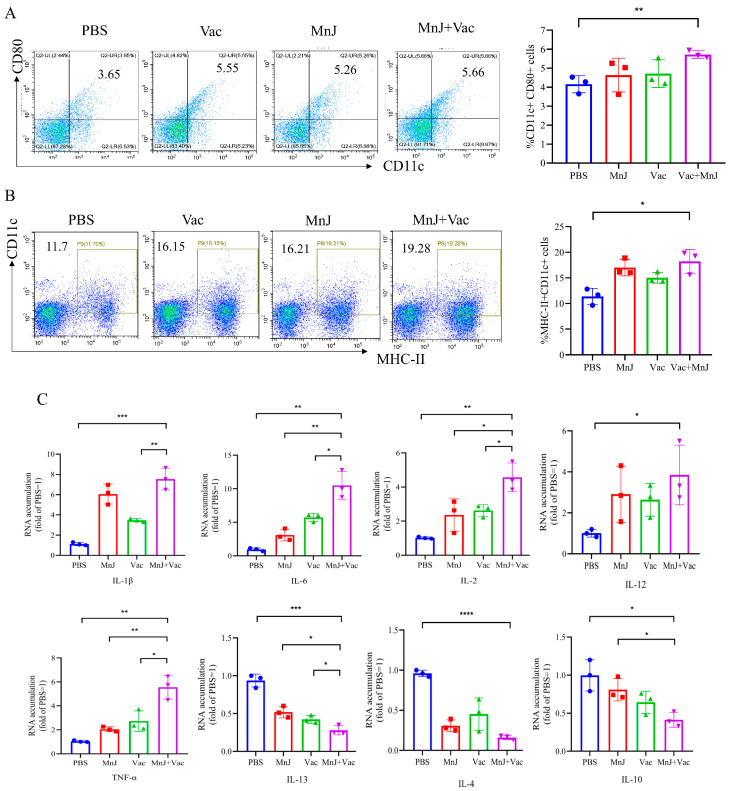
MnJ adjuvant promoted DC maturation. ICR mice were immunized via the intramuscular injection route (*n* = 3) with PBS, MnJ adjuvant, VAC, VAC + MnJ. At 3 days post-inoculation (dpi), lymph nodes (LNs) were collected, and single cells were stained with CD11c, CD80, and MHC-II antibodies. Cellular debris was excluded using a forward scatter area (FSC-A) vs. side scatter area (SSC-A) dot plot to obtain total cells; single cells were gated on a forward scatter height (FSC-H) vs. forward scatter width (FSC-W) dot plot; and CD11c^+^MHC-II^+^ and CD11c^+^CD80^+^ cell populations were gated, with thresholds set by isotype controls. (**A**) Representative flow cytometry plots of CD11c^+^ CD80^+^ cDCs in the LNs of the four groups and statistical results of the proportion of CD11c^+^ CD80^+^ cDCs. (**B**) Representative flow cytometry plots of MHC-II^+^ CD11c^+^ cDCs in the LNs of the four groups and statistical results of the proportion of MHC-II^+^ CD11c^+^ cDCs. (**C**) RNA was extracted from the lymph nodes, and quantitative real-time polymerase chain reaction (qRT-PCR) analysis was performed. * *p* < 0.05; ** *p* < 0.01; *** *p* < 0.001; **** *p* < 0.0001.

To investigate whether MnJ adjuvant could enhance the dendritic cell (DC) maturation in mice, DCs were isolated from the mice’s lymph nodes 3 days after immunization, and then analyzed by flow cytometry. The level of CD11c^+^ (a traditional marker for cDC in mice) CD80^+^ (a DC maturation marker) was 5.66% in group D (VAC + MnJ), which was higher than group B (VAC) (5.55%) (Figure 2A). The MHC-II^+^ CD11c^+^ level reached 19.28% in group D, while that of group B was 16.15%. (Figure 2B). From the results we could find that MnJ adjuvant promoted the maturation of cDCs (CD11c^+^ CD80^+^ cDCs).

The expression level of cytokines in the lymph nodes was exanimated by qPCR. The expression levels of IL-1β, IL-6, IL-2, TNF-α and IL-12 were higher in group D compared to other groups (Figure 2C). Moreover, the levels of IL-13 and IL-4 in group D decreased significantly (Figure 2C), and the level of IL-10 also decreased significantly (Figure 2C). The significant increase in pro-inflammatory factors and decrease in anti-inflammatory factors and immunoregulatory factors indicated a strong Th1-type response was induced.

### 3.2. MnJ Adjuvant Enhanced Splenocyte Proliferation and IFN-γ Secretion

**Figure 3:**
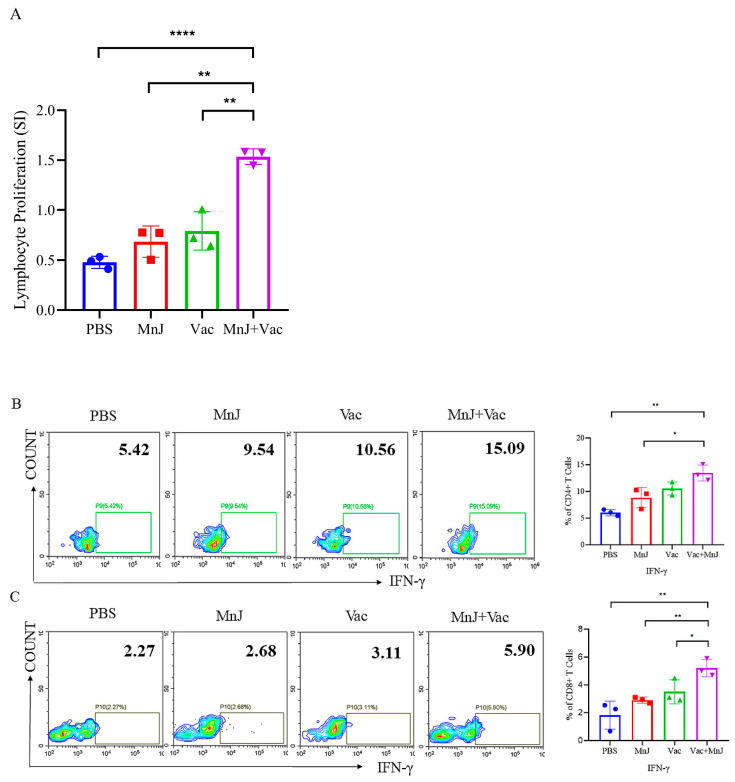
MnJ adjuvant enhanced splenocyte proliferation and IFN-γ secretion. ICR mice were immunized via the intramuscular injection route (*n* = 3) with PBS, MnJ adjuvant, VAC, and VAC + MnJ. A total of three immunizations were carried out, with an interval of 2 weeks between each immunization. At 35 days post-inoculation (dpi) (7 days after the last immunization), the spleens were collected, and splenocytes were stimulated with TLA. Splenocyte supernatant was collected, and single cells were stained with CD3, CD4, CD8 and IFN-γ antibodies. Debris exclusion via FSC-A vs. SSC-A; T cells were gated as CD3^+^ cells on FSC-A vs. CD3 dot plot; CD4^+^ T cells and CD8^+^ T cells were further discriminated within the CD3^+^ population on a CD4 vs. CD8 dot plot. IFN-γ^+^ cells were identified within CD3^+^ CD4^+^ T cells, with thresholds set by isotype controls. (**A**) Proliferative activity of splenic lymphocytes. (**B**) Representative flow cytometry plots of CD4^+^ IFN-γ^+^ cells in the splenocytes of the four groups and statistical results of the proportion of CD4^+^ splenocytes producing IFN-γ. (**C**) Representative flow cytometry plots of CD8^+^ IFN-γ^+^ cells in the splenocytes of the four groups and statistical results of the proportion of CD8^+^ splenocytes producing IFN-γ. * *p* < 0.05; ** *p* < 0.01; **** *p* < 0.0001.

Splenocytes were collected 7 days after the last immunization, and treated with TLA and ConA. The proliferation was analyzed by CCK8, and the responses of CD4^+^ and CD8^+^ T cells producing IFN-γ were analyzed by flow cytometry. The results showed that the lymphocyte proliferation SI of group D was significantly higher than other groups (Figure 3A). The proportion of cells producing IFN-γ in the splenocytes of group D was significantly higher than other groups (Figure 3B,C), indicating that MnJ adjuvant enhanced the immunogenicity of VAC through the promotion of IFN-γ production.

### 3.3. MnJ Adjuvant Enhanced the Generation of Memory T Cells

**Figure 4:**
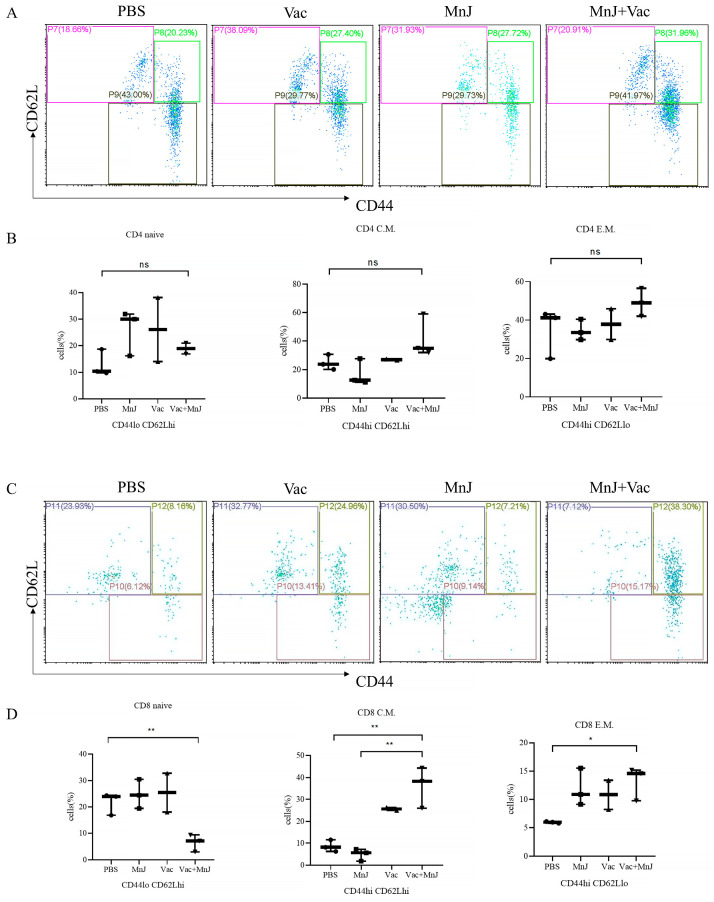
MnJ adjuvant enhanced the generation of memory T cells. ICR mice were immunized via the intramuscular injection route (*n* = 3) with PBS, MnJ adjuvant, VAC, and VAC + MnJ. Three immunizations were carried out in total, with an interval of 2 weeks between each immunization. At 35 days post-inoculation (dpi) (7 days after the last immunization), the spleens were collected, and splenocytes were stimulated with TLA. Splenocyte supernatant was collected, and single cells were stained with CD3, CD4, CD8, CD44 and CD62L antibodies. (**A**) Representative flow cytometry plots of CD4^+^ memory T cells in the splenocytes of the four groups. (**B**) The statistical results of the proportion of CD4^+^ memory T cells (CD4 naive: CD4^+^ CD44^lo^CD62L^hi^, CD4 C.M.: CD4^+^ CD44^hi^CD62L^hi^, CD4 E.M.: CD4^+^ CD44^hi^CD62L^lo^). (**C**) Representative flow cytometry plots of CD8^+^ memory T cells in the splenocytes of the four groups (CD8 naive: CD8^+^ CD44^lo^CD62L^hi^, CD8 C.M.: CD8^+^ CD44^hi^CD62L^hi^, CD8 E.M.: CD8^+^ CD44^hi^CD62L^lo^). (**D**) The statistical results of the proportion of CD8^+^ memory T cells. * *p* < 0.05; ** *p* < 0.01; ns, no significant difference.

To further investigate the induction of memory T cells post-vaccination, splenocytes were harvested from mice 7 days following the final immunization and subsequently stimulated in vitro with TLA. The frequency of memory T cells was detected via flow cytometry, and group D exhibited elevated levels of CD8^+^ central memory T cells (TCM) and CD8^+^ effector memory T cells (TEM) (Figure 4D). There was no significant difference in CD4^+^ central memory T cells and CD4^+^ effector memory T cells between group D and the other groups (Figure 4C). The MnJ adjuvant effectively promotes the differentiation of CD8^+^ T cells into the memory state, which may improve the durability of immunity and the ability to respond to reinfection.

### 3.4. MnJ Adjuvant Promoted Th1 Immune Responses

**Figure 5:**
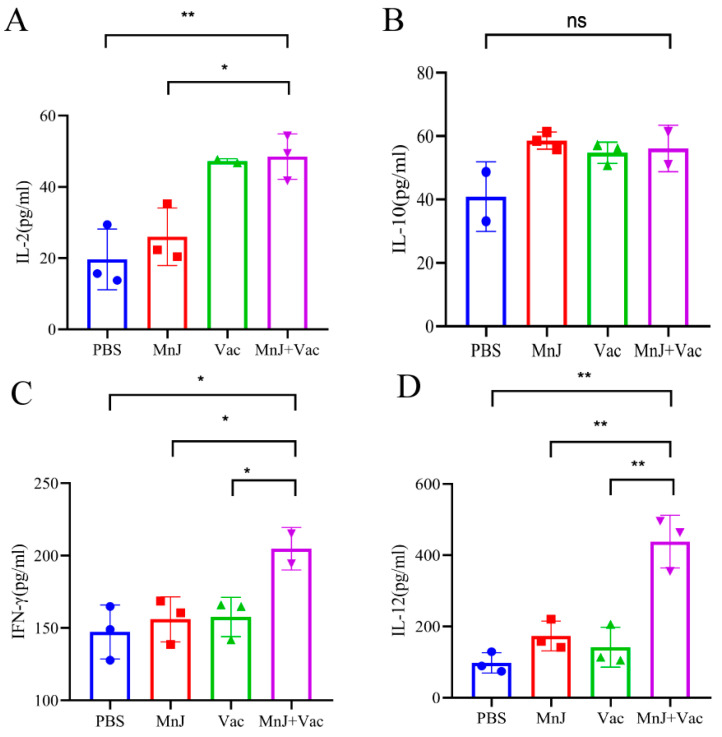
MnJ adjuvant promoted Th1 immune responses. ICR mice were immunized via the intramuscular injection route (*n* = 3) with PBS, MnJ adjuvant, VAC, and VAC + MnJ. A total of three immunizations were performed, with an interval of 2 weeks between each immunization. At 35 days post-inoculation (dpi) (7 days after the last immunization), the spleens were collected, and splenocytes were stimulated with TLA. The supernatants were collected and used to detect the cytokines IL-2 (**A**), IL-10 (**B**), IL-12 (**C**), and IFN-γ (**D**) by ELISA. * *p* < 0.05; ** *p* < 0.01; ns, no significant difference.

To evaluate the secretion of cytokines including IL-2, IL-12, IFN-γ and IL-10, the supernatant was harvested from stimulated splenocytes explained in Section 3.3. From the results we found that the levels of IL-2, IL-12 and IFN-γ in group D were significantly higher than other groups, while no significant difference was found in IL-10 (Figure 5A–D).

### 3.5. MnJ Adjuvant Promoted Humoral Immune Responses

**Figure 6:**
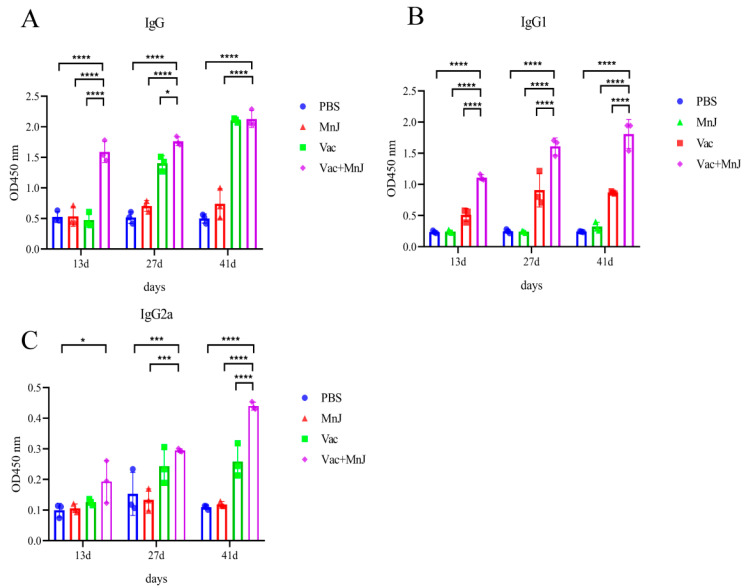
MnJ adjuvant promoted Th1 immune responses. ICR mice were immunized via the intramuscular injection route (*n* = 3) with PBS, MnJ adjuvant, VAC, and VAC + MnJ. A total of three immunizations were performed, with an interval of 2 weeks between each immunization. Blood samples were collected at 13, 27, and 41 dpi, and IgG (**A**), IgG1 (Th2) (**B**), and IgG2a (Th1) (**C**) were detected by ELISA. * *p* < 0.05; *** *p* < 0.001; **** *p* < 0.0001.

To evaluate the *T. gondii*-specific antibody response, serum samples were collected from each group 13, 27 and 41 days after immunization, and the total IgG and IgG subclasses (IgG1 and IgG2a) were analyzed by ELISA. The levels of serum-specific IgG, IgG1 and IgG2a antibodies in the mice of groups C and D showed an upward trend with the increase in immunization time and frequency. High levels of IgG, IgG1 and IgG2a were observed in the serum on the 41st day (Figure 6A–C).

### 3.6. MnJ Adjuvant Increased the Protection Against Acute and Chronic T. gondii Infections in Mice

**Figure 7:**
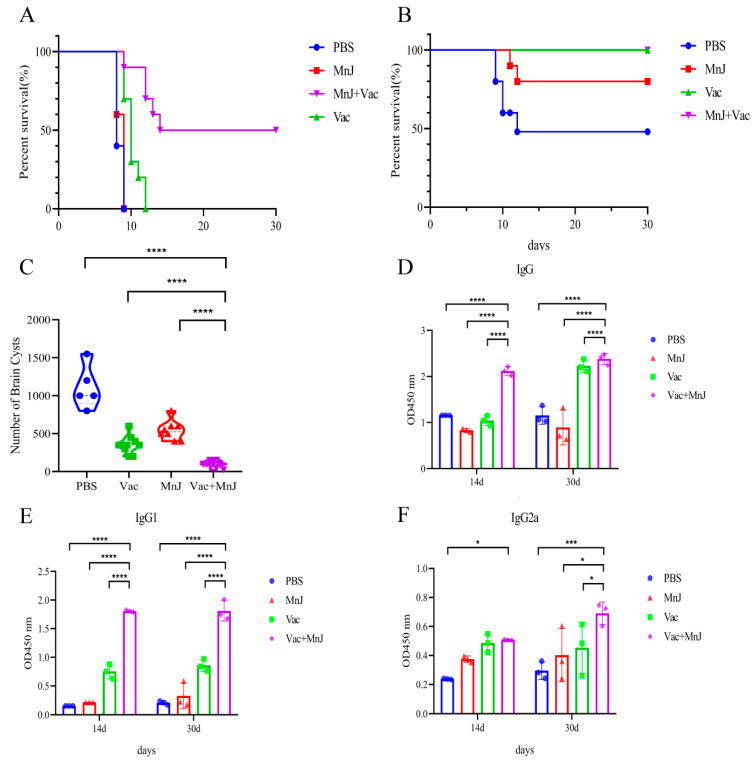
MnJ adjuvant increased the protection against acute and chronic *T. gondii* infections in mice. Kaplan–Meyer plots displaying survival over time of different treatments/immunizations: (**A**) Survival curves of mice challenged intraperitoneally with 1 × 10^2^ tachyzoites of the *T. gondii* RH strain. (**B**) Protection efficiency of different groups against chronic infection was evaluated by cyst burden assays. (**C**) The number of brain cysts in the four groups of mice after chronic infection. The mice were chronically infected, and blood samples were collected at 14 and 30 days post-infection (dpi). IgG (**D**), IgG1 (**E**), and IgG2a (**F**) were detected by ELISA. * *p* < 0.05; *** *p* < 0.001; **** *p* < 0.0001.

To evaluate the acute protective effect, each mouse was challenged with 1 × 10^2^ RH tachyzoites (*i.p.*), and the survival situation was recorded every day. Survival rates of different groups are shown in Figure 7A. Group D (VAC + MnJ) (50%) was significantly higher than other groups. The incorporation of MnJ adjuvant significantly enhanced the efficacy of VAC in improving mice’s survival rate, resulting in a 50% survival rate.

To further evaluate the protective effect against chronic *T. gondii* infection, 10 mice from each group were orally infected with 20 cysts. After 30 days, the mice were sacrificed and brain cysts were calculated. The survival rate of the mice in group A (PBS) was 50%, that of group C (MnJ) was 80%, and that of groups B (VAC) and D (VAC + MnJ) was 100% (Figure 7B). The number of brain cysts in group D was significantly reduced (71.2%) comparing to group B, and reduced 90.77% compared to group A (Figure 7C). This reduction indicated that MnJ adjuvant significantly improved the protection of the vaccine against latent parasitic persistence.

Three mice per group were bled on the 14th and 30th days after chronic challenge to detect the levels of *T. gondii*-specific antibodies (Figure 7D–F). High serum titers of IgG, IgG1, and IgG2a were maintained post-chronic challenge in group D.

### 3.7. The Incorporation of MnJ Adjuvant Promoted a Faster and Stronger Immune Response Against Acute T. gondii Infection

**Figure 8:**
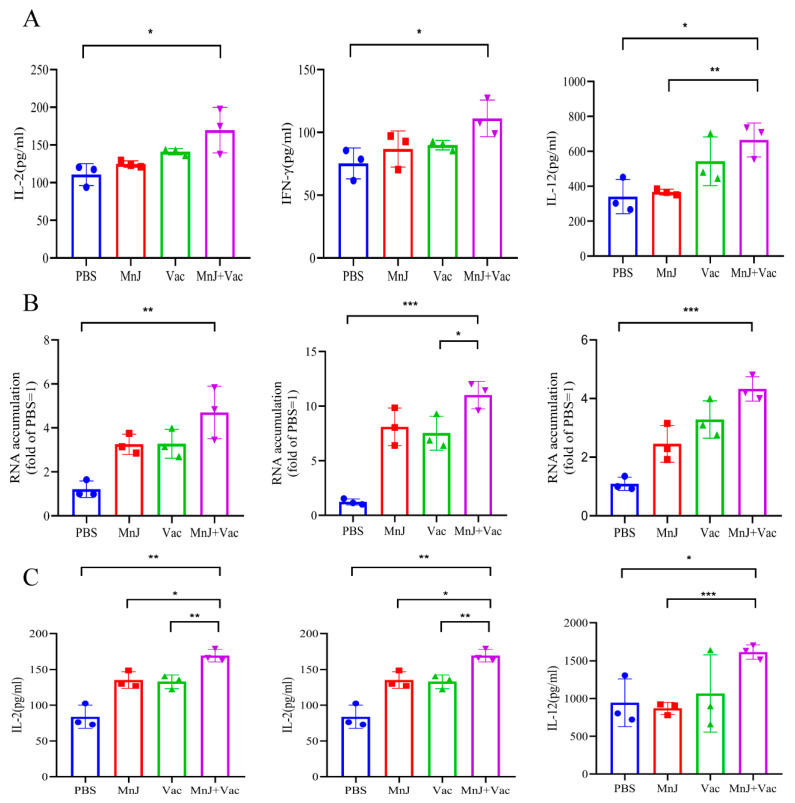
The incorporation of MnJ adjuvant promoted a faster and stronger immune response against acute *T. gondii* infection. ICR mice were immunized via the intramuscular injection route with PBS, MnJ adjuvant, VAC, and VAC + MnJ. A total of three immunizations were conducted, with an interval of 2 weeks between each immunization. At 42 days post-inoculation (dpi), each mouse was intraperitoneally injected with 100 tachyzoites of the *T. gondii* RH strain. Blood samples were collected and spleens were harvested at 45 dpi. (**A**) Splenocytes were stimulated with TLA, and the supernatants were collected for the detection of cytokines by ELISA. (**B**) RNA was extracted from the splenocytes, and quantitative real-time polymerase chain reaction (qRT-PCR) analysis was performed. (**C**) Serum cytokines were detected by ELISA. * *p* < 0.05; ** *p* < 0.01; *** *p* < 0.001.

Blood samples were collected and splenocytes were isolated from the mice on the 3rd day after acute challenge. The splenocytes were stimulated by TLA, and the levels of IL-2, IL-12, and IFN-γ were examined; from the results we found that the cytokines were higher than other groups (Figure 8A). The mRNA expression levels of these three cytokines (IL-2, IL-12, IFN-γ) were detected in the spleen by qPCR, which showed a consistent trend with the protein levels (Figure 8B). For the blood samples, the levels of IL-2, IL-12, and IFN-γ in group D were significantly higher than other groups (Figure 8C). These results demonstrated that the activation of Th1-type cytokines (IL-2, IL-12, IFN-γ) was enhanced by MnJ adjuvant following acute *T. gondii* challenge, thereby eliciting robust cellular immune responses.

## 4. Discussion

Inactivated vaccines, characterized by stable physicochemical properties and low biosafety risks, have become the preferred choice for industrial production [8]. However, the inactivation process destroys the metabolic activity of pathogens, making it difficult to effectively stimulate cellular immunity, and their immunoprotective efficacy is far lower than that of attenuated vaccines [17]. Therefore, breaking through the immunogenicity bottleneck of inactivated vaccines through technical means such as developing new adjuvants and optimizing antigen delivery systems has become a core research direction in *Toxoplasmosis* control.

This study innovatively combines the VAC with MnJ adjuvants and exhibits significant advantages compared to traditional inactivated vaccines. The addition of MnJ adjuvants enhance the immune response at multiple levels. Specifically, in the early stage of immune activation, MnJ adjuvants can rapidly promote the maturation of DCs, which, as the most potent antigen-presenting cells in the immune system, play a crucial role in initiating immune responses and regulating the types of immune reactions [18]. Conventional mature dendritic cells (cDCs) upregulate the costimulatory molecules CD80 and/or CD86, along with major histocompatibility complex class II (MHC-II) [19,20,21]. This cellular event is critical for facilitating the crosstalk between cDCs and T cells. Our experimental results showed that CD11c^+^CD80^+^ of DCs in the lymph nodes of mice induced by MnJ adjuvant and VAC was significantly upregulated, which indicated that more antigen-presenting cells possess the capacity to activate T cells, allowing the immune response to be initiated rapidly and laying the foundation for the subsequent proliferation, differentiation and effector functions of T cells.

Consistently with cDC maturation phenotype, the secretion of pro-inflammatory cytokines IL-1β, IL-6, TNF-α, and IL-12 in the lymph nodes was increased. As a key pro-inflammatory cytokine, IL-1β can activate immune cells, trigger inflammatory responses, and subsequently initiate the host’s immune defense mechanism [22]. IL-6 exerts a critical regulatory effect on the activation and proliferation of immune cells [23]. TNF-α exerts extensive immunomodulatory functions and can enhance the cytotoxic activity of immune cells [24]. IL-12 plays an indispensable role in promoting the activation and differentiation of T cells and natural killer (NK) cells, as well as assisting in the polarization of the Th1-type immune response [25]. In this study, we found that the levels of Th2-type anti-inflammatory factors IL-13 and IL-4 and immune regulatory factor IL-10 decreased. As a key immunomodulatory factor, IL-10 primarily functions to inhibit the excessive activation of immune cells and sustain the homeostasis of the immune system [26]. The decrease further indicates that MnJ adjuvant altered the host’s original immune balance, shifting the immune response toward a robust Th1-type response. All the aforementioned changes laid the foundation for cellular immunity-dominated anti-*T. gondii* immunity. Notably, we found that qRT-PCR analysis conducted in lymph nodes demonstrated a significant reduction in the mRNA level of the immunomodulatory factor IL-10, thereby supporting Th1 immune polarization; in contrast, ELISA analysis of supernatants derived from stimulated splenocyte cultures revealed no significant differences in IL-10 protein secretion levels among the groups. This observed discrepancy may be attributed to variations in detection time points and sample types: lymph node samples were harvested on day 3 after the first immunization (i.e., the early phase of innate immune activation), whereas splenocyte supernatants were collected on day 7 following the final immunization (i.e., the peak phase of adaptive immune responses). Collectively, these findings suggest that MnJ adjuvant significantly suppresses this key immune regulatory factor during the early activation phase (within lymph nodes); however, following the establishment of adaptive immunity (in the spleen), IL-10 secretion may be rebalanced by other cell populations to preserve immune homeostasis. We found that the lymph node proliferation index (SI) in group D was significantly higher than that in the other groups, indicating that the adjuvant enhanced the vaccine’s capacity to induce *T. gondii*-specific immune cells in the spleen and thus confirming the ability to activate the host’s cellular immune system. In the core process of cellular immunity, the proliferation of T lymphocytes was significantly enhanced, and the generation of CD8^+^ memory T cells was increased, providing long-term immune protection for the body and enabling a rapid response when *T. gondii* invades again. In terms of humoral immunity, the levels of specific IgG, IgG2a, and IgG1 antibodies in group D were significantly increased, enhancing the ability of antibodies to clear *T. gondii* post-infection.

Our research results showed that after *T. gondii* RH tachyzoite challenge, the survival rate of mice in the MnJ adjuvant and VAC group reached 50%, which was significantly higher than that of the control groups. This protection was also much better than protein vaccines and DNA vaccines [27,28]. After chronic cyst challenge, all mice in group D survived; the number of brain cysts decreased significantly (90.77%), and a relatively high titer of specific antibodies was maintained. The combination of the MnJ adjuvant and the VAC helps to improve the durability of immunity. Furthermore, given that IFN-γ and IL-12 play a crucial role in limiting the proliferation of tachyzoites in the early stage of *T. gondii* infection, our findings demonstrated that the levels of IL-2, IL-12, and IFN-γ produced by splenocytes in group D and VAC on the third day after RH challenge were significantly higher than those in the PBS group on protein and mRNA levels, which confirmed that the incorporation of the MnJ adjuvant significantly enhanced the VAC’s capacity to elicit robust and rapid immune responses.

Interestingly, although group D (VAC + MnJ) exhibited significantly enhanced Th1 immune responses in most indicators, several key findings warrant in-depth interpretation. First, at the early stage of immune activation, there was only a minimal difference in the level of the lymph node dendritic cell (DC) maturation marker CD11c+CD80+ between the VAC-alone group (group B) and the VAC + MnJ group (group D) (5.55% vs. 5.66%), indicating that the inactivated vaccine (VAC) itself possesses a certain capacity to activate DCs, and the main role of MnJ adjuvant may not lie in increasing the magnitude of basal DC activation but rather in regulating subsequent immune signaling pathways and polarization; this requires further verification. Second, MnJ adjuvant exhibited no significant effect on the generation of CD4^+^ memory T cells, which may be associated with the primary mechanism of action of MnJ, thereby specifically enhancing CD8^+^ T cell-mediated memory differentiation.

## 5. Conclusions

This study represents the first documented evaluation of MnJ adjuvant as an immunoadjuvant in an inactivated *T. gondii* vaccine formulation. The results of the study demonstrate that MnJ significantly enhances innate and adaptive immunity, particularly cellular immunity in a mouse model. Notably, the incorporation of MnJ adjuvant resulted in a 50% survival rate in mice during acute *T. gondii* infection and significantly reduced cerebral cyst burden in chronic toxoplasmosis models. These findings provide a foundational framework for optimizing adjuvant selection for parasitic diseases.

## Figures and Tables

**Table 1:** Primers of the cytokines and regulatory factors used for quantitative real-time PCR.

Primer	Sequence (5′ to 3′)
IL-10	GCTCTTGCACTACCAAAGCC
	CTGCTGATCCTCATGCCAGT
IL-2	ACTCACCAGGATGCTCACAT
	AGACTTGTCTACCTAATGGA
IL-12	TTGATGATGACCCTGTGCCT
	GTGATTCTGAAGTGCTGCGT
IL-13	AGCATGGTATGGAGTGTGGA
	TTGCAATTGGAGATGTTGGT
IL-4	AACAAGGAACACCACGGAGAA
	TCAAGCACGGAGGTACATCAC
IFN-γ	GGATGCATTCATGAGTATTGC
	CCTTTTCCGCTTCCTGAGG
TNF-α	GGTGCCTATGTCTCAGCCTCT
	GCCATAGAACTGATGAGAGGG
IL-1β	TGGACCTTCCAGGATGAGG
	GTTCATCTCGGAGCCTGTAGT
IL-6	TACCACTTCACAAGTCGGAGG
	CTGCAAGTGCATCATCGTTGTT

## Data Availability

The data presented in this study are available from the corresponding author upon reasonable request.
